# (*S*)-α-Chlorohydrin Inhibits Protein Tyrosine Phosphorylation through Blocking Cyclic AMP - Protein Kinase A Pathway in Spermatozoa

**DOI:** 10.1371/journal.pone.0043004

**Published:** 2012-08-20

**Authors:** Hao Zhang, Huan Yu, Xia Wang, Weiwei Zheng, Bei Yang, Jingbo Pi, Gengsheng He, Weidong Qu

**Affiliations:** 1 Key Laboratory of the Public Health Safety, Ministry of Education, Department of Environmental Health, School of Public Health, Fudan University, Shanghai, China; 2 Neurology Department of Huashan Hospital, Fudan University, Shanghai, China; 3 Institutes for Chemical Safety Sciences, The Hamner Institutes for Health Sciences, Research Triangle Park, North Carolina, United States of America; 4 Department of Histology and Embryology, Basic Medical College, China Medical University, Shenyang, China; Laurentian University, Canada

## Abstract

α-Chlorohydrin is a common contaminant in food. Its (*S*)-isomer, (*S*)-α-chlorohydrin (SACH), is known for causing infertility in animals by inhibiting glycolysis of spermatozoa. The aim of present work was to examine the relationship between SACH and protein tyrosine phosphorylation (PTP), which plays a critical role in regulating mammalian sperm capacitation. In vitro exposure of SACH 50 µM to isolated rat epididymal sperm inhibited PTP. Sperm-specific glyceraldehyde 3-phosphate dehydrogenase (GAPDS) activities, the intracellular adenosine 5′-triphosphate (ATP) levels, 3′-5′-cyclic adenosine monophosphate (cAMP) levels and phosphorylation of protein kinase A (PKA) substrates in rat sperm were diminished dramatically, indicating that both glycolysis and the cAMP/PKA signaling pathway were impaired by SACH. The inhibition of both PTP and phosphorylation of PKA substrates by SACH could be restored by addition of cAMP analog dibutyryl-cAMP (dbcAMP) and phosphodiesterase inhibitor 3-isobutyl-1-methylxanthine (IBMX). Moreover, addition of glycerol protected glycolysis, ATP levels, phosphorylation of PKA substrates and PTP against the influence of SACH. These results suggested SACH inhibited PTP through blocking cAMP/PKA pathway in sperm, and PTP inhibition may play a role in infertility associated with SACH.

## Introduction

α-Chlorohydrin (3-chloro-1,2-propanediol, ACH) is a well-known food contaminant, usually formed during acid-catalyzed hydrolysis of vegetable proteins [1], [2] or in domestic cooking [3]. This contaminant is found in various food products such as soy sauce [4], bread, biscuits, cheese and bacon [5], [6] as well as drinking water treated with epichlorohydrin resins [7]. It is reported recently that ACH esters are often present in refined oil at very high levels, e.g. 1–10 mg/kg, which is thousands times higher than that of ACH in soy sauce [8]–[10]. Free ACH can be released from ACH esters in the gastrointestinal tract [10].

ACH can cause a reversible male infertility. For instance, ACH exposure rapidly causes rapid infertility in various mammalian species, including rats [11], rams [12], boars [13], and monkeys [14] but not mouse [15] after oral exposure at relatively low doses for a brief period. This sterility is accompanied by reduced sperm motility while testicular spermatogenesis remains normal [16], [17]. ACH possesses a chiral carbon atom and the antifertility effect is attributed to the (*S*)-isomer [18]. Within sperm, (*S*)-α-chlorohydrin (SACH) is metabolized to (*S*)-3-chlorolactaldehyde [19], which selectively inhibits sperm specific glyceraldehyde 3-phosphate dehydrogenase (GAPDS) [20], [21], and blocks glycolysis pathway [22]. Glycolysis is thought to be one of the major resources of sperm energy [23]–[25]. Thus the blocking of glycolysis by SACH results in deficiency of adenosine 5′-triphosphate (ATP) and impairment of sperm motility. However, it had been pointed out that other sperm functions might be impaired by SACH. For instance, Jelks et al. [26] reported that sperm obtained from ACH treated rats failed to fertilize oocytes in vitro even though ACH was given at the doses not affecting sperm motility.

In mature spermatozoa, the nucleus is highly compacted in head and sperm are almost transcriptionally silent and translationally inactive [27], so sperm rely more on post-translational modification, especially protein tyrosine phosphorylation (PTP) to regulate their physiological processes. PTP plays a significant role in regulation of sperm function including capacitation, hyperactivation and acrosome reaction [28], [29]. 3′-5′-cyclic adenosine monophosphate (cAMP)/protein kinase A (PKA) pathway has been identified as the principle way of PTP regulation [29]–[31]. In this pathway, cAMP is predominantly synthesized by ‘soluble’ adenylyl cyclase (sAC), an atypical adenylyl cyclase activated mainly by bicarbonate and Ca^2+^ but insensitive to G proteins [32], [33], and degraded by phosphodiesterases (PDEs). PKA is stimulated by cAMP to phosphorylate specific substrates on serine (Ser) and threonine (Thr) residues and then the downstream signaling cascades are subsequently up-regulated and finally PTP increases [34].

There is increasing evidence that glucose metabolism is required for sperm PTP [24], [35], [36]. Mouse sperm in media absent of glucose are unable to complete PTP [37], [38], and glycolytic inhibitors ornidazole and oxamate inhibited PTP in hamster or mouse sperm, respectively [39], [40]. With respect to the close relationship between glycolysis and PTP, we suspect that SACH might affect PTP in rat sperm. Moreover, it still remains unclear that how glucose metabolites play a role in PTP or how glycolytic inhibitors compromise PTP. To answer these questions will help to understand the molecular processes of SACH's adverse effects on male fertility, and also give an insight into the relationship between energy metabolism and regulatory mechanism of capacitation in mammalian sperm. In the current study, we started our work with characterizing PTP patterns of rat sperm exposed to SACH while under capacitating conditions and then attempted to explore the mechanism of SACH inhibition PTP. Our results provided direct evidence that the SACH inhibited PTP through suppression of cAMP/PKA signal pathway which resulted from blocking of glycolysis.

## Results

### SACH inhibits tyrosine protein phosphorylation in rat spermatozoa

**Figure 1:**
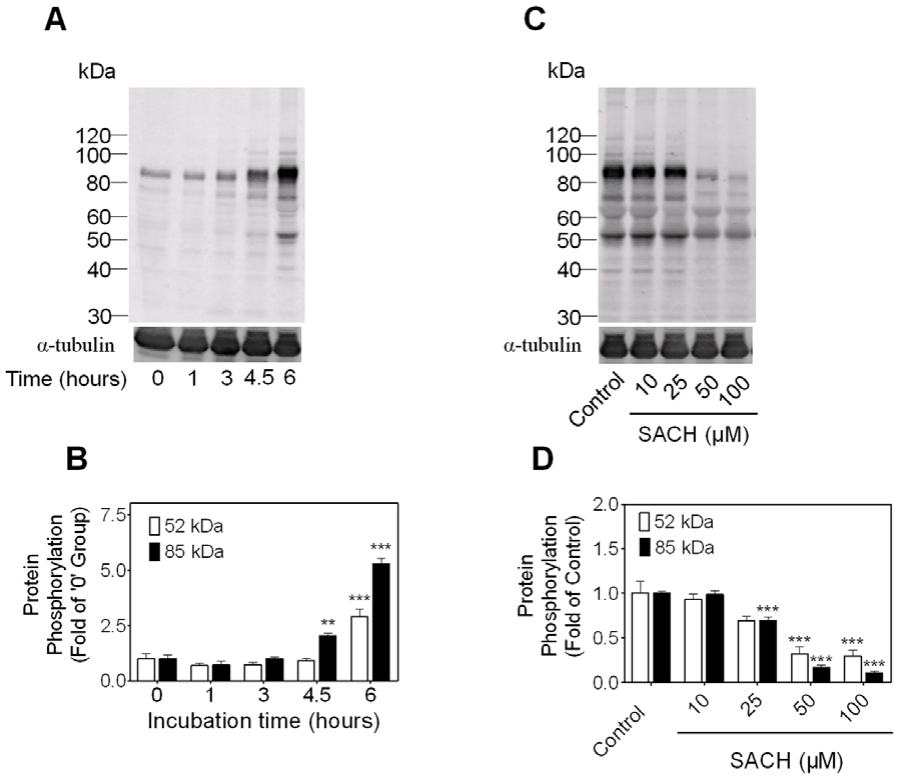
SACH inhibits capacitation-associated PTP in rat caudal epididymal spermatozoa. (A) A time course of increases of PTP in rat sperm during capacitation. Sperm were incubated in BWW at 37°C for 0, 1, 3, 4.5 and 6 hours, and cell lysates subjected to SDS-PAGE and Western Blotting with anti-PTP antibody as described in Materials and methods. The molecular mass (kDa) of the protein standard is indicated on the left. (B) Quantification of PTP levels of 52 kDa and 85 kDa bands of four independent immunoblots of Fig. 1 A and data are presented as mean ± SEM. n = 4, ** *p*<0.01, *** *p*<0.001vs. 0 hour group. (C) SACH inhibited PTP in rat caudal sperm in a dose dependent pattern. Sperm were exposed to SACH at the indicated concentrations for 6 hours in BWW at 37°C. (D) Relative intensities of bands of 52 kDa and 85 kDa in Fig. 1 C, n = 4, * *p*<0.01, *** *p*<0.001 vs. control.

Capacitation is a time dependent process and PTP is recognized as a hallmark of capacitation [31], [41], and various periods of time are required to achieve PTP for different species. As shown in Fig. 1A, an increase of tyrosine phosphorylation of a subset of proteins of 40–220 kDa occurred in rat epididymal sperm over a 6 hour incubation time under capacitating conditions. Taking the bands of 52 kDa and 85 kDa for example, the PTP states of these proteins had increased by 5–6 folds at the end of the incubation (Fig. 1B).

The effects of in vitro exposure to SACH for 6 hours on PTP are shown in Fig. 1C and Fig. 1D. PTP was inhibited by SACH in a concentration-related pattern. Although SACH reduced rat sperm kinematic parameters (Fig. S1), it seems not kill sperm at the concentration of 100 µM, because the median lethal concentration (LC_50_) of SACH for rat sperm was about 100 mM (Fig. S2).

### The role of cAMP-PKA pathway in the inhibition of protein tyrosine phosphorylation induced by SACH

**Figure 2:**
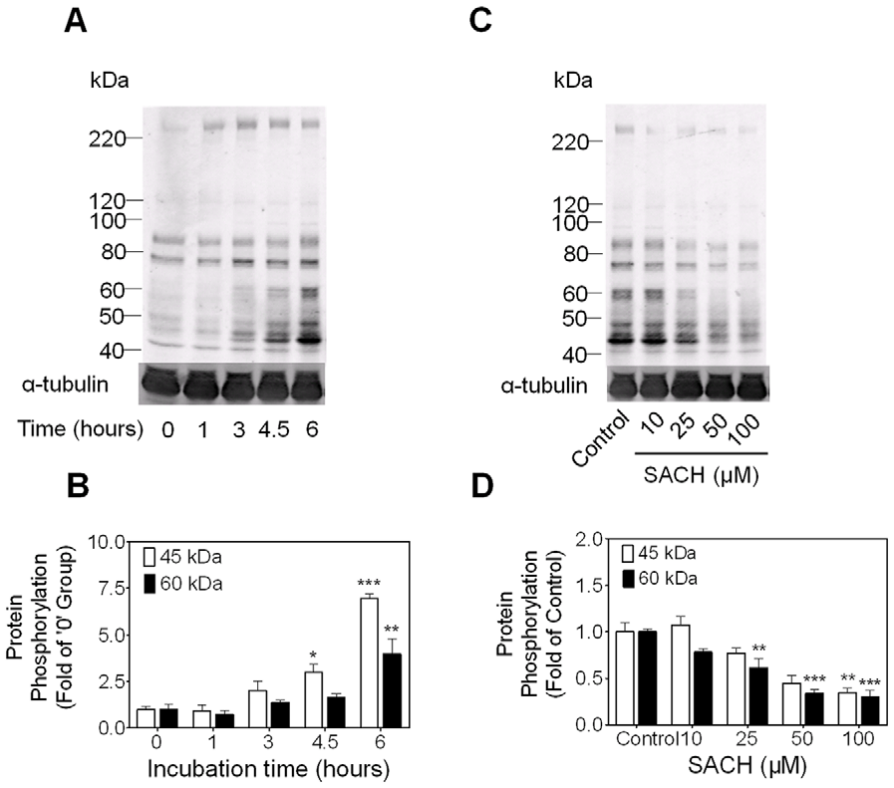
SACH inhibits P-PKAs, which increases in rat caudal sperm during capacitation. (A and B) Rat sperm were incubated in BWW at 37°C for 0, 1, 3, 4.5 and 6 hours, before immunoblotting of P-PKAs. Mean ± SEM of triplicate independent experiments are shown, * *p*<0.05, ** *p*<0.01, *** *p*<0.001 vs. 0 hour group. (C and D) Rat sperm were exposed to various concentrations of SACH for 6 hours in capacitating condition and immunoblotted with anti-P-PKAs antibody. n = 3, * *p*<0.05, ** *p*<0.01, *** *p*<0.001 vs. control.

As mentioned before, PTP in sperm is principally regulated by a cAMP-dependent PKA pathway. To investigate whether this pathway was affected by SACH we assayed the phosphorylation of PKA substrates (P-PKAs) of rat sperm after incubation in capacitating medium in the presence or absent of SACH. A time-dependent elevation of P-PKAs (45 kDa – >220 kDa) during the capacitation was observed (Fig. 2A, 2B). When treated with SACH, a concentration–dependent reduction of P-PKAs indicated that PKA activity declined in the presence of SACH (Fig. 2C, 2D).

**Figure 3:**
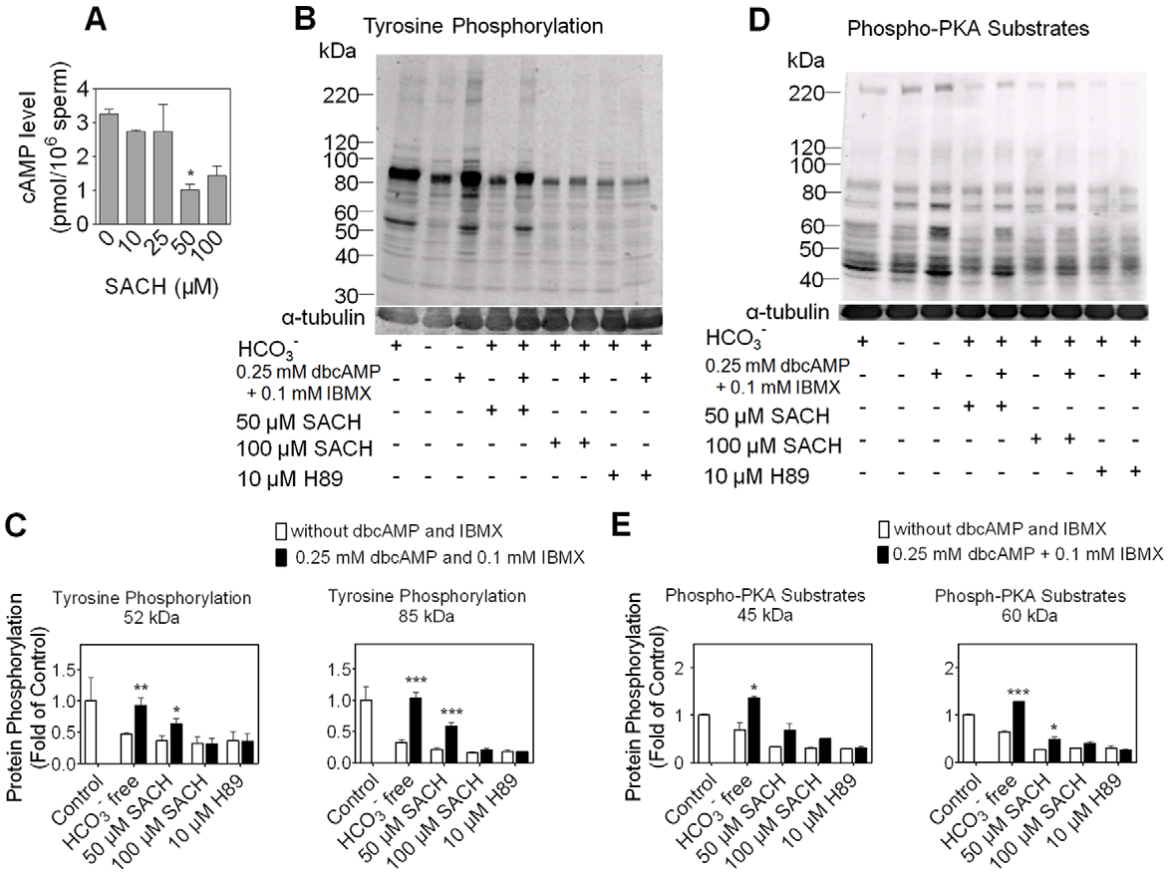
The role of depletion of cAMP in inhibition of PTP and P-PKAs by SACH. (A) cAMP levels in rat sperm decreased by SACH after incubation in capacitating conditions for 6 hours, * *p*<0.05 vs. control. (B C, D and E) Rat sperm were incubated in complete BWW supplemented with 50 µM SACH, 100 µM SACH and 10 µM H89 or in BWW-HCO_3_^−^ in the presence and absence of 0.25 mM dbcAMP and 0.1 mM IBMX as indicated at the bottoms of the figures. After 6 hours of incubation, PTP (B) and P-PKAs (D) were identified by Western Blot, and levels of phosphorylation were quantitated by densitometry analysis of representative bands (C, E). n = 3, * *p*<0.05, ** *p*<0.01, *** *p*<0.001 vs. control.

PKA is regulated by cAMP level. As PKA activity was not directly affected by SACH (Fig. S3), then we assayed cAMP level in rat sperm treated with various concentration of SACH in vitro. Fig. 3A shows that 50 µM and 100 µM SACH significantly reduced cAMP level in sperm, implying that the decrease of cAMP might be involved in the depression of PTP.

Since SACH exposure reduced cAMP content and P-PKAs in rat sperm, we tried to use the cAMP analogue dbcAMP together with the PDE inhibitor IBMX to restore the suppression of capacitation-associated phosphorylation. As shown in Fig. 3B (lane 2) and Fig. 3D (lane 2), the medium free of HCO_3_^−^, a prime activator of sAC, could not support PTP and P-PKAs in rat sperm, but both of them were restored by the addition of dbcAMP and IBMX (Fig. 3B lane 3; Fig. 3D lane 3). On the other hand, addition of dbcAMP and IBMX failed to restore the inhibition of P-PKAs and PTP caused by H89, a PKA inhibitor (Fig. 3B lane 8, 9; Fig. 3D lane 8, 9). These data confirmed that PTP was controlled by cAMP-dependent PKA pathway in rat sperm, and dbcAMP and IBMX could substitute cAMP to activate PKA but could not prevent the direct inhibition of PKA. Therefore, addition of dbcAMP and IBMX blocked the inhibition of PTP and P-PKAs caused by 50 µM SACH (Fig. 3B lane 4, 5; Fig. 3D lane 4, 5). However, they had little ability to alter the inhibition of phosphorylation caused by 100 µM SACH (Fig. 3B lane 6, 7; Fig. 3D lane 6, 7). Together with cAMP data, the results suggested that the deficiency of cAMP could, at least in part, explain the inhibition of the phosphorylation in capacitation.

### The role of glycolysis in the inhibition of protein tyrosine phosphorylation by SACH

**Figure 4:**
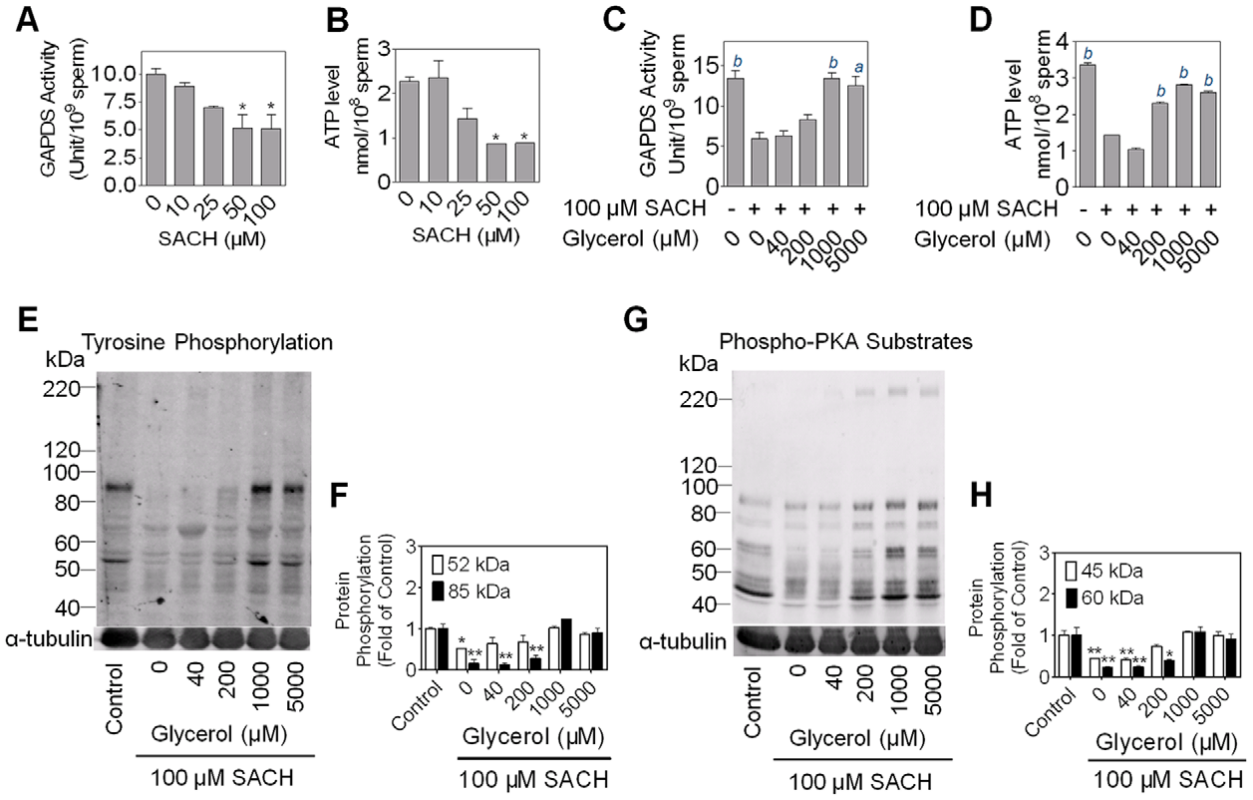
Glycerol rescues glycolysis, PTP and PKA after inhibition by SACH. Rat spermatozoa were exposed to a series of concentrations of SACH in capacitating conditions for 6 hours and both GAPDS activity (A) and ATP level (B) were significantly reduced. n = 3, * *p*<0.05 vs. control. (C and D) Rat sperm were exposed to a serial of concentrations of glycerol in the presence of 100 µM SACH for 6 hours and GAPDS activity and ATP were measured. n = 3, *a p*<0.01, *b p*<0.001 vs. the 100 µM SACH group. (E, F, G and H) With the same treatment as in (C and D), sperm were subjected to Western Blot analyses with anti-PTP (E and F) and anti-P-PKAs (G and H) antibodies. n = 2, * *p*<0.05, ** *p*<0.01 vs. control.

SACH diminishes sperm energy production by blocking glycolysis. As expected, 50 µM or higher concentration of SACH reduced GAPDS activity (Fig. 4A) and whole cell ATP levels (Fig. 4B). Glycerol is reported to block the transformation of ACH to 3-chlorolactaldehyde in mammalian sperm [42]. Thus we used glycerol to test the relation between glycolysis and the cAMP/PKA pathway. When different concentrations of glycerol were added to rat sperm in the presence of SACH, it restored GAPDS activity, ATP levels, PTP and P-PKAs to normal levels as the glycerol concentration increased (Fig. 4C, 4D, 4E and 4G). This restoration by glycerol indicated that the blockade of glycolysis was a key element in impairment of the cAMP/PKA pathway in sperm.

## Discussion

It is well established that PTP of sperm is a critical event in fertilization. Although the identification of proteins that undergoing tyrosine phosphorylation during capacitation in rat sperm still remains unknown, proteomics methodologies such as 2-dimensional gel electrophoresis and tandem mass spectrometry (MS/MS) have been applied to identification of PTP in human [43], hamster [44], boar [45], mouse [46] and buffalo and cattle sperm [47]. It had been reported that in human sperm ion channels, calcium binding protein CABYR [48], and metabolic enzymes and structural proteins [43] were tyrosine-phosphorylated during capacitation.

Few toxicological investigations have studied this essential factor. Our data demonstrate that SACH inhibits PTP in rat epididymal sperm. PTP in human ejaculated sperm was found sensitive to SACH as well (data now shown). Furthermore, similar results in rhesus macaque sperm had been observed when the sperm were exposed to 0.5 mM ACH in vitro [49]. These findings indicated that SACH might affect sperm capacitation through interrupting signaling events. In addition, the inhibition of TPT did not result from change of sperm viability, because rat sperm remained relative good viability in a medium with SACH no more than 10 mM. The viability of rat sperm treated with 0.1, 1.0 and 10 mM SACH was 82.1–88.5% of the control (Fig. S2). In another study, the percentages of vital sperm incubated in the same concentrations of ACH were 36.0–44.4%, while the control was 48.6% [50].

As far as we know, there was no comprehensive study on identification of the target proteins of PKA in sperm, nevertheless it is speculated that PKA may module tyrosine kinases or tyrosine phosphatases activities by phosphorylating their Ser/Thr residues [51]. We demonstrate that SACH inhibits P-PKAs which increase in a time-dependent manner in rat sperm under capacitating condition. The increase of P-PKAs is known to occur within a few minutes after mouse [52], [53], boar [54] and human [55] sperm are released into bicarbonate-containing medium. However, in the present study P-PKAs in rat sperm developed in a much slower pattern matching with the process of PTP, which is considered as ‘slow capacitation events’ [30], [56]–[57], indicating that rat sperm regulate capacitation in a some different way. However, sperm of different species, including rat, may share the HCO_3_^—^sAC–cAMP pathway to regulate P-PKAs [57]. The depression of P-PKAs and cAMP levels indicate SACH negatively regulate PTP through reduction of cAMP levels and subsequent PKA activity.

Clearly, blocking of glycolysis plays a pivotal role in SACH inhibition of this capacitation signal pathway. Previous studies have shown ACH affected sperm GAPDS activity or ATP levels [22], [58]–[59]. In this study, on only GAPDS activity and ATP levels, but also cAMP levels, P-PKAs and PTP significantly decreased while rat sperm were exposed to SACH. Glycerol strongly antagonized SACH induced glycolysis blockade, which is consistent with what founded in boar sperm [22], while restored levels of ATP, P-PKAs and PTP. Although sperm could utilize glycerol to synthesized ATP [60], it does not appear that ATP derived from glycerol compensated for energy loss from blocked glycolysis, because in rat sperm the rate of glycerol metabolism was only 1.2%–3.6% of the rate of glucose metabolism [61]. Furthermore, glycerol is metabolized through glycolytic pathway [62]–[63], so sperm could not use glycerol to produce ATP if GAPDS had been inhibited by SACH or its metabolite. Antagonistic action of glycerol against SACH might be attributed to glycerol competitively inhibited a NADP+ dependent dehydrogenase which metabolizes SACH into (S)-3-chlorolactaldehyde. This hypothesis is consistent with the fact that rat sperm remained their motility when exposed to both 1.0 mM glycerol and 100 µM SACH (Fig. S4), while glycerol failed to restore rat sperm motility which was inhibited by SACH in vivo (data not shown).

As the alteration of cAMP and ATP levels are essential to the inhibition of PTP, it is necessary to elucidate how the depletion of ATP led to a deficiency in cAMP. sAC may be the connecting point between these events. In mammalian sperm ATP is used as the substrate of sAC to synthesis to cAMP. But the affinity of sAC for ATP is 10-fold lower than the transmembrane adenylyl cyclase [64], and the Michaelis-Menten constant (*K_m_*) of sAC for ATP-Mg^2+^ has been reported at 10 mM [33], or 16.1 mM [65]. Despite the abundance of ATP in mammalian sperm cytoplasm, as for instance, the 15 mM concentration of ATP in rat sperm [66] and 20 mM in bull sperm [67], these levels are close to sAC's *K_m_*, which means the velocity of sAC reaction could be sensitive to variation in ATP concentrations. Thus sAC could function as a cellular energy sensor [68]. It is possible that cAMP level in sperm, together with downstream evens, may be stalled by the change of ATP levels when glycolysis is inhibited by SACH. However, in the case of PKA, which also requires ATP to transfer phosphate to its protein substrates, the *Km* of PKA is about 40 µM [56] or 200 µM ATP [53] which is sufficient to support PKA to phosphorylate its substrates in vitro. Therefore, it was not likely that SACH caused ATP levels to become too low to become able to induce PKA phosphorylation.

It should be noted that dbcAMP and IBMX failed to resort PTP or P-PKAs inhibited by 100 µM SACH, which implies that other factors besides cAMP depletion might be involved in SACH's action. The states of P-PKAs was balanced by the activities of PKA and Ser/Thr phosphatases, and the later play a critical role in regulation of P-PKAs during sperm capacitation [56]. Several lines of evidence demonstrate that glucose metabolism in sperm is linked to production of reactive oxygen species (ROS) [28], [69], which is considered as an activator of Ser/Thr phosphatases [70]–[71], so it is possible that the blocking of glycolysis led to depletion of ROS and reduced the activities of Ser/Thr phosphatases, which, or perhaps combining with cAMP depletion, resulted in the inhibition of P-PKAs and subsequent PTP.

**Figure 5:**
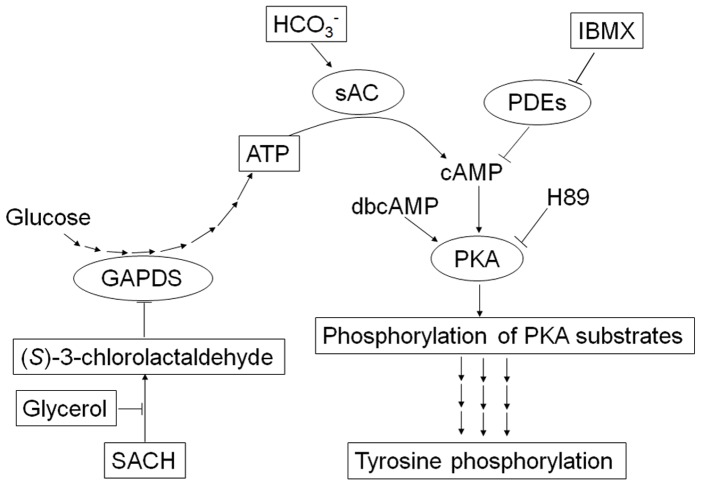
The model by which SACH inhibits cAMP/PKA mediated protein phosphorylation through blocking glycolysis in spermatozoa during capacitation. The inhibition of GAPDS by SACH results in depletion of ATP and then depresses of P-PKAs and PTP. Glycerol overcomes these effects through restoring glycolysis, and IBMX and dbcAMP, the activators of PKA, reverse in part the inhibitions of P-PKAs and PTP.

In summary, our data demonstrated that SACH inhibited phosphorylation of PKA substrate and tyrosine residues through impairing energy generation from glycolysis and subsequent deficiency of cAMP level and PKA inactivation (Fig. 5). Considering this study is only based on in vitro experiments, further work is needed to see if long term environmental exposure to ACH will lead to impairment of male fertility or diminishment of PTP. Nevertheless, based on the susceptibility of sperm PTP and the strong association between PTP and human semen quality [72]–[74], It is prudent that PTP or other molecular markers in this signaling pathway could be developed to be a valuable endpoint for assessment of male reproductive risks of chemicals exposure such as ACH.

## Materials and Methods

### Ethics Statement

Animal ethics approval was obtained from Fudan University of Animal Ethics Committee. All procedures were conducted according to the National Ethics Committee for Care and Use of Laboratory Animals for Research of the Medical School.

### Reagents and materials

Bovine serum albumin fraction V (BSA), Nicotinamide adenine dinucleotide (NAD^+^), 3-[(3-cholamidopropyl) dimethylammonio]-1-propanesulfonate (CHAPS), Dithiothreitol (DTT), Percoll, 4-(2-hydroxyethyl)-1-piperazineethanesulfonic acid (HEPES), Tween 20, Tris base, potassium fluoride, glucose, pyruvate, lactate, glycerol, penicillin, streptomycin, glyceraldehyde-3-phosphate, sodium pyrophosphate, sodium arsenate, oxalate, oxamate, β-mercaptoethanol, aprotinin, leupeptin and anti-α-tubulin antibody were obtained from Sigma- Aldrich (St. Louis, MO, USA). N-[2-(p-Bromocinnamylamino)ethyl]-5-isoquinolinesulfonamide (H89), 3-isobutyl-1-methylxanthine (IBMX) and dibutyryl-cAMP (dbcAMP) were acquired from Merck (Darmstadt, Germany). The methyl tetrazolium (MTT) assay system was purchased from Beyotime Institute of Biotechnology. Antibodies anti-phospho-tyrosine mouse mAb (P-Tyr-100) and anti-phospho-(Ser/Thr) PKA substrate were purchased from Cell Signaling Technology (Danvers, MA, USA).

### Animals

Retired Spague-Dawley male rats were purchased from Shanghai Laboratory Animal Center, Chinese Academy of Science (Shanghai, China), and maintained five per cage in a condition with constant temperature (22±2°C) and humidity (50%), and light/dark cycle (12 hours each).

### Culture media

The basic medium used for capacitating rat sperm was a modified Biggers Whitten & Whittingham (BWW) medium [75] consisting of 94.6 mM NaCl, 4.8 mM KCl, 1.2 mM MgSO_4_, 1.2 mM NaH_2_PO_4_, 5.6 mM glucose, 0.25 mM sodium pyruvate, 21.6 mM sodium lactate, 1.7 mM CaCl_2_, 25.1 mM NaHCO_3_, 10 mM HEPES (pH 7.5), and 15 mg/ml of BSA. BWW free of bicarbonate (Non-capacitation medium) was prepared by substituting NaHCO_3_ with 25.1 mM NaCl to maintain osmosis.

### Rat sperm collection and incubation

Adult male rats were sacrificed by CO_2_ asphyxiation and cauda epididymis were minced swiftly in 1.5 ml pre-warmed BWW medium without bicarbonate. After 5 min incubation in 37°C, 5% CO_2_/95% air, sperm suspension was diluted equably into proper media at final concentration of 2–4×10^6^ cell/ml and incubated in 37°C, 5% CO_2_/95% air for 6 hours. Especially, if the time course of PTP during capacitation was to be set up, the sperm were collected immediately when diluted into BWW medium (time = 0 hour), and then collected at the time points of 1 hour, 3 hour, 4.5 hour and 6 hour. Sperm counting were performed with a 0.1 mm depth hemacytometer.

### Measurement of Sperm Kinematics

At the end of incubation or treatment, a measurement of rat sperm kinematics was performed a Computer Assisted Sperm Analysis (CASA) (TOX IVOS, Hamilton Thorne Research, Inc., Beverly, MA, USA). The following parameters were measured: motile, curvilinear velocity (VCL), average path velocity (VAP), straight line velocity (VSL), amplitude of lateral head movement (ALH), beat cross frequency (BCF) and straightness (STR). At least 200 sperm were analyzed for each measured.

### MTT assay

Briefly, sperm were incubated for 2 hours with diluted MTT solution according to the products instruction, and then Formanzan Addition of Solubilization Solution was followed by about 4 hours incubation until solid formanzan was completely resolved. Absorbance was recorded at 560 nm.

### SDS-PAGE and Western Blot

At the end of incubation, the sperm were collected and centrifuged at 7,000×g, 4°C for 5 min, washed 3 times with cold phosphate buffered saline (PBS), and resuspended in Laemmli sample buffer without β-mercaptoethanol and boiled for 5 min. And then the samples were centrifuged at 10,000×g, 4°C for 5 min. The supernatants were removed into a new tube and added final concentration of 5% β-mercaptoethanol then boiled for 5 min. Sperm protein extracted from 1×10^6^ sperm were loaded to 10% SDS-PAGE gels for electrophoresis and then transferred to PVDF membranes (Millipore, Bedford, MA) at 45 volts for 3 hours. After blocking with 3% BSA in Tris buffered saline with 0.1% Tween 20 (TBST) at 4°C overnight, the membranes were incubated with first antibodies for 2 hours at room temperature at following dilutions in TBST: anti-Phospho-Tyrosine (P-Tyr-100) 1∶2000, anti-phospho-PKA substrates 1∶1000, anti-α-tubulin 1∶5000. Washing the membranes with TBST for 3 times was followed by blotting with second antibodies conjugated with horseradish peroxidase for 2 hours. Proteins were visualized using enhanced chemiluminescence detection kit (ECL plus, Amersham Biosciences). When necessary, the membranes were stripped with stripping buffer and reprobed other target proteins. Western blot intensity was quantitated with ImageJ software (http://rsb.info.nih.gov/ij/).

### Measurement of GAPDS activity

Rat sperm were collected and washed with cold PBS after treatment with or without SACH, discarded the supernatant by aspiration. 600 µl sonication buffer consisted of 0.3% HCAPS, 150 mM NaCl, 1 mM DTT, 10 µg/ml aprotinin and 10 µg/ml leupeptin was added and the suspension was sonicated 3 times on ice. GAPDS enzyme reaction took place in a mixture described by Welch et al. [76] which containing 0.25 mM NAD, 3.3 µM DTT, 0.3 mM glyceraldehyde-3-phosphate, 5 mM potassium fluoride, 0.5 mM oxalate, 15 mM sodium pyrophosphate, and 30 mM sodium arsenate, 0.1 mM oxamate, and the change of absorbance at 340 nm was read immediately.

### PKA activity assay

Sperm PKA activity was measured using a nonradioactive PKA assay kit (V5340, Promega Corp., Madison, WI, USA). Rat sperm were washed twice with cold PBS and homogenized on ice in 0.5 ml PKA Extraction Buffer. After centrifugation at 14,000×g for 5 min at 4°C, the supernatant was removed. The reaction system contained 5 µl PepTag PKA reaction buffer, 5 µl PepTag A1 peptide, 5 µl cAMP, 1 µl peptide protection solution, and 4 µl sample homogenate, and added H_2_O to a final volume of 25 µl. The reaction was carried out for 30 min at room temperature, and stopped by 5 min's boiling. The phosphorylated peptides were separated by electrophoresis on a 0.8% agarose gel at 100 V for 15 min. Excised the negatively charged phosphorylated bands from the gel, heated at 95°C, resolved the hot agarose with 75 µl of Gel Solubilization Solution and 50 µl of glacial acetic acid, quantitated by the absorbance at 570 nm on spectrophotometry. One unit of PKA activity was defined as the number of nanomoles of phosphate transferred to a substrate per minute per 10^7^ sperm.

### ATP assay

ATP concentrations were analyzed using a bioluminescent ATP assay kit (FLASC, Sigma). In brief, 2×10^5^ rat sperm were collected and washed with cold PBS after 6 hours incubation, intracellular ATP was released by the manufacturer-supplied lysis buffer. Sample ATP extractions and a serial of ATP standard were mix with luciferase reagent and the luminescence was read immediately.

### cAMP assay

The DetectX cAMP Chemiluminescent Immunoassay kits (Arbor Assays, Ann Arbor, MI, USA) were used to measure rat sperm cAMP level. According to the instruction of the kit, 1×10^6^ rat sperm were collected at the end of incubation, and washed with PBS by centrifuging at 5000×g, 4°C for 15 min followed by lysis and acetylation. Luminescence was read by 96 well microplate reader (Molecular Devices SpectraMax M5, USA). Acetylation reagents, namely triethylamine and acetic anhydride, are lachrymators and should be used in hood.

### Statistics

The values were expressed as means ± standard error of the mean (SEM). All graphs or analyses were constructed or performed using GraphPad Prism (GraphPad Software, San Diego, CA, USA). Comparisons between the experimental groups were analyzed using one-way ANOVA followed by Dunnett's Multiple Comparison test with the significance level set at P<0.05.

## Supporting Information

Figure S1 **Effects of SACH on rat sperm motility.** Rat sperm were incubated with 10, 25, 50 and 100 µM SACH for 6 hours in BWW, 37°C and then sperm kinematic parameters Motile, VSL, VAP, VCL, ALH, LIN, BCF and STR were measured using CASA system. Each point represents mean ±SEM, n = 3. * P<0.05 *vs*. control.
(TIF)

Figure S2 **Cytotoxicity of SACH on rat sperm.** Rat sperm were incubated in BWW in the presence of 0.01, 0.1, 1, 10, 100 and 1000 mM SACH for 6 hours and then a methyl tetrazolium (MTT) assay was performed. Each point presents as mean ± SEM, n = 4.
(TIF)

Figure S3 **Effects of SACH on PKA activity in rat sperm extracts.** Rat sperm were homogenized and the cytoplasm extracts were incubated with 10, 25, 50 and 100 µM SACH in PKA reaction mixture for 30 min, and then followed by PKA activity measurement. The reaction system without PKA was taken as negative control, and the positive control reaction system contained 0.4 µg/ml PKA catalytic subunit. Data represent as mean ± SEM, n = 3.
(TIF)

Figure S4 **Glycerol restores rat sperm motility inhibited by SACH.** Rat sperm were treated with 0, 0.04, 0.2 and 1.0 mM glycerol in the presence of 100 µM SACH for 6 hours in BWW, and CASA was performed to measure sperm kinematic parameters motile, VSL, VAP, VCL, ALH, LIN, BCF and STR. Data represent as mean ± SEM, n = 3.
(TIF)
