# Supplementary figures and images for: (S)-α-Chlorohydrin Inhibits Protein Tyrosine Phosphorylation through Blocking Cyclic AMP - Protein Kinase A Pathway in Spermatozoa
Source: PLoS One. 2012 Aug 20;7(8):e43004. doi: 10.1371/journal.pone.0043004 (PMC3423423; doi:10.1371/journal.pone.0043004)

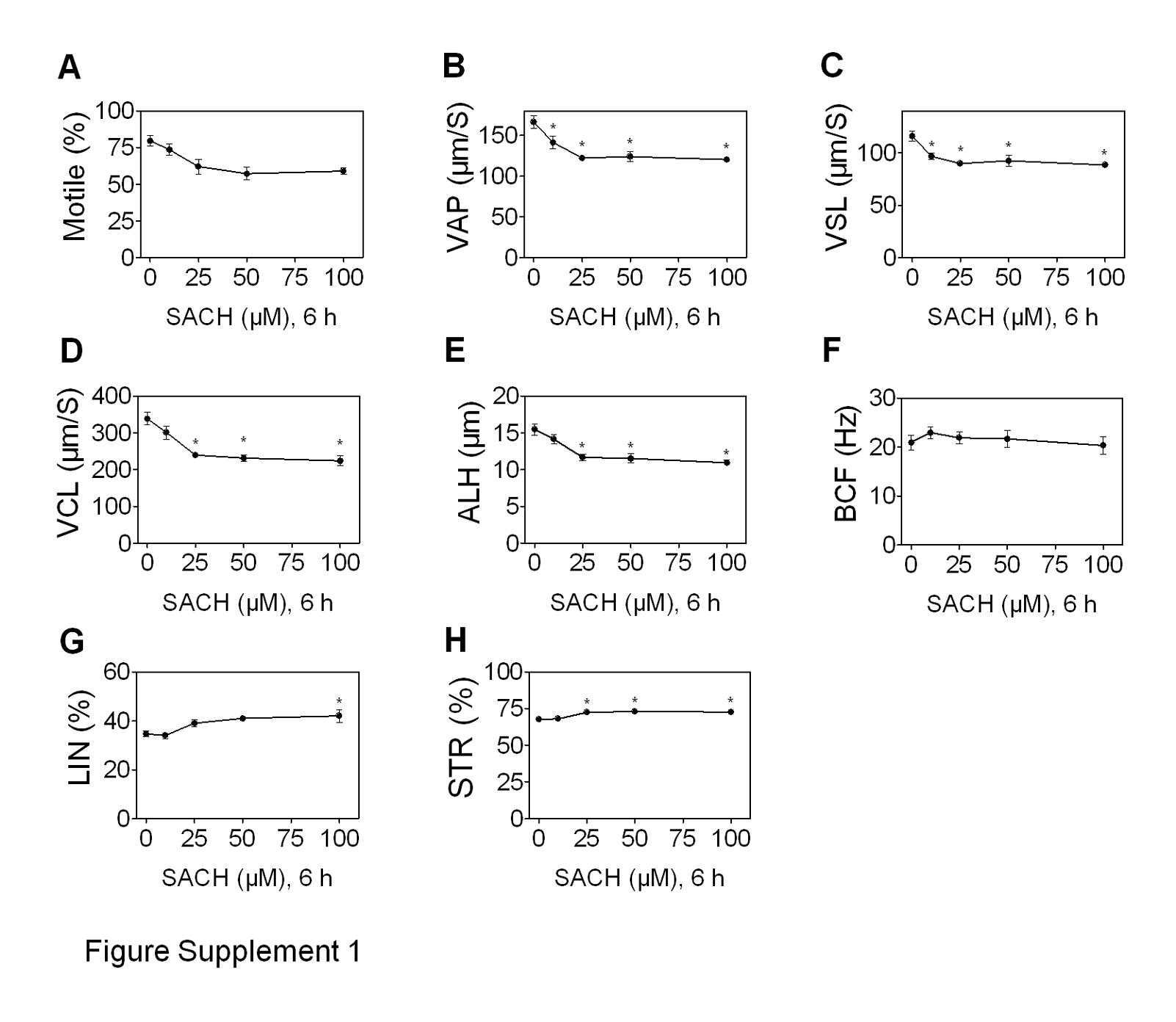

Supplement: Figure S1 — Effects of SACH on rat sperm motility. Rat sperm were incubated with 10, 25, 50 and 100 µM SACH for 6 hours in BWW, 37°C and then sperm kinematic parameters Motile, VSL, VAP, VCL, ALH, LIN, BCF and STR were measured using CASA system. Each point represents mean ±SEM, n = 3. * P<0.05 vs. control. (TIF) [file pone.0043004.s001.tif]

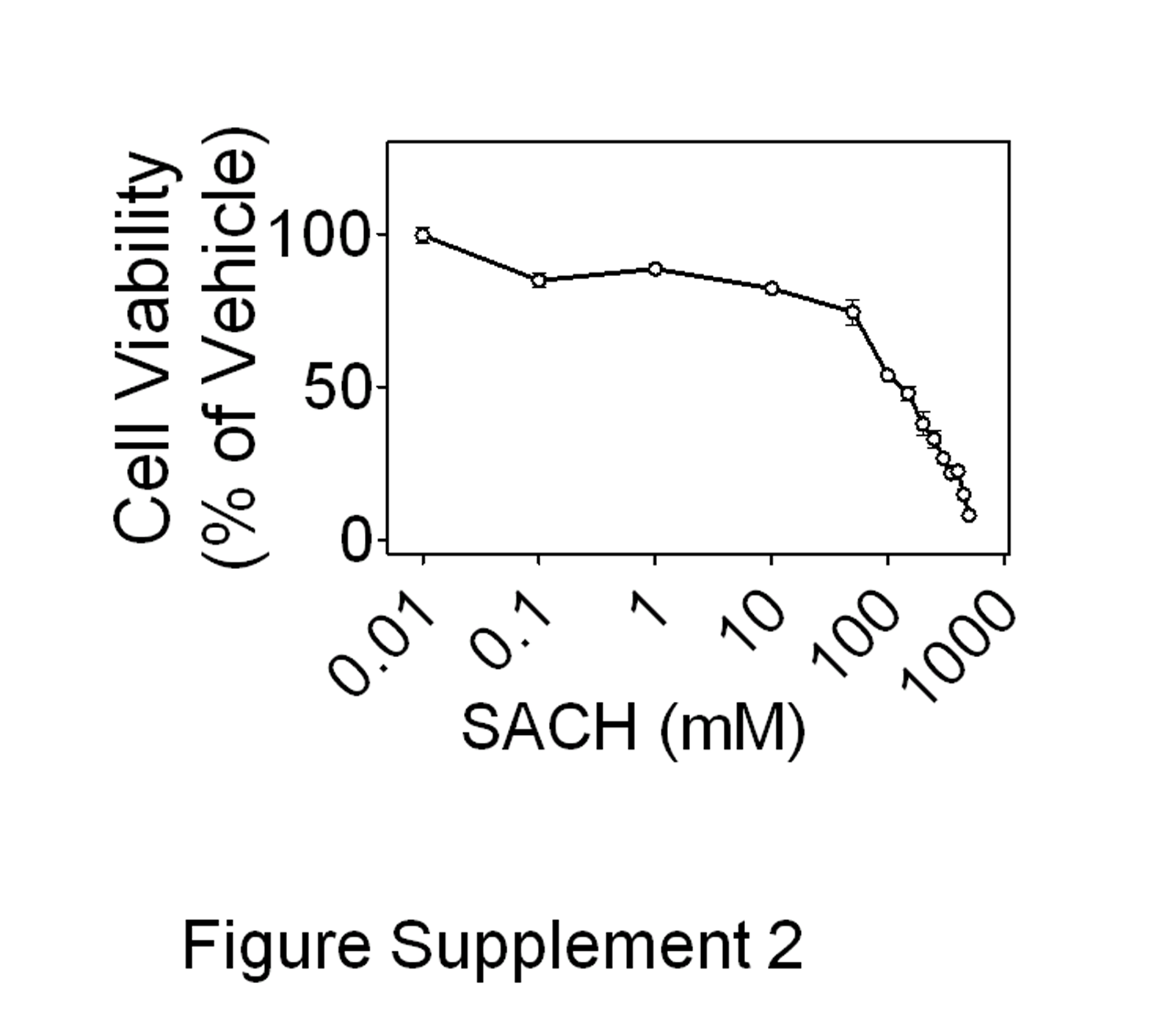

Supplement: Figure S2 — Cytotoxicity of SACH on rat sperm. Rat sperm were incubated in BWW in the presence of 0.01, 0.1, 1, 10, 100 and 1000 mM SACH for 6 hours and then a methyl tetrazolium (MTT) assay was performed. Each point presents as mean ± SEM, n = 4. (TIF) [file pone.0043004.s002.tif]

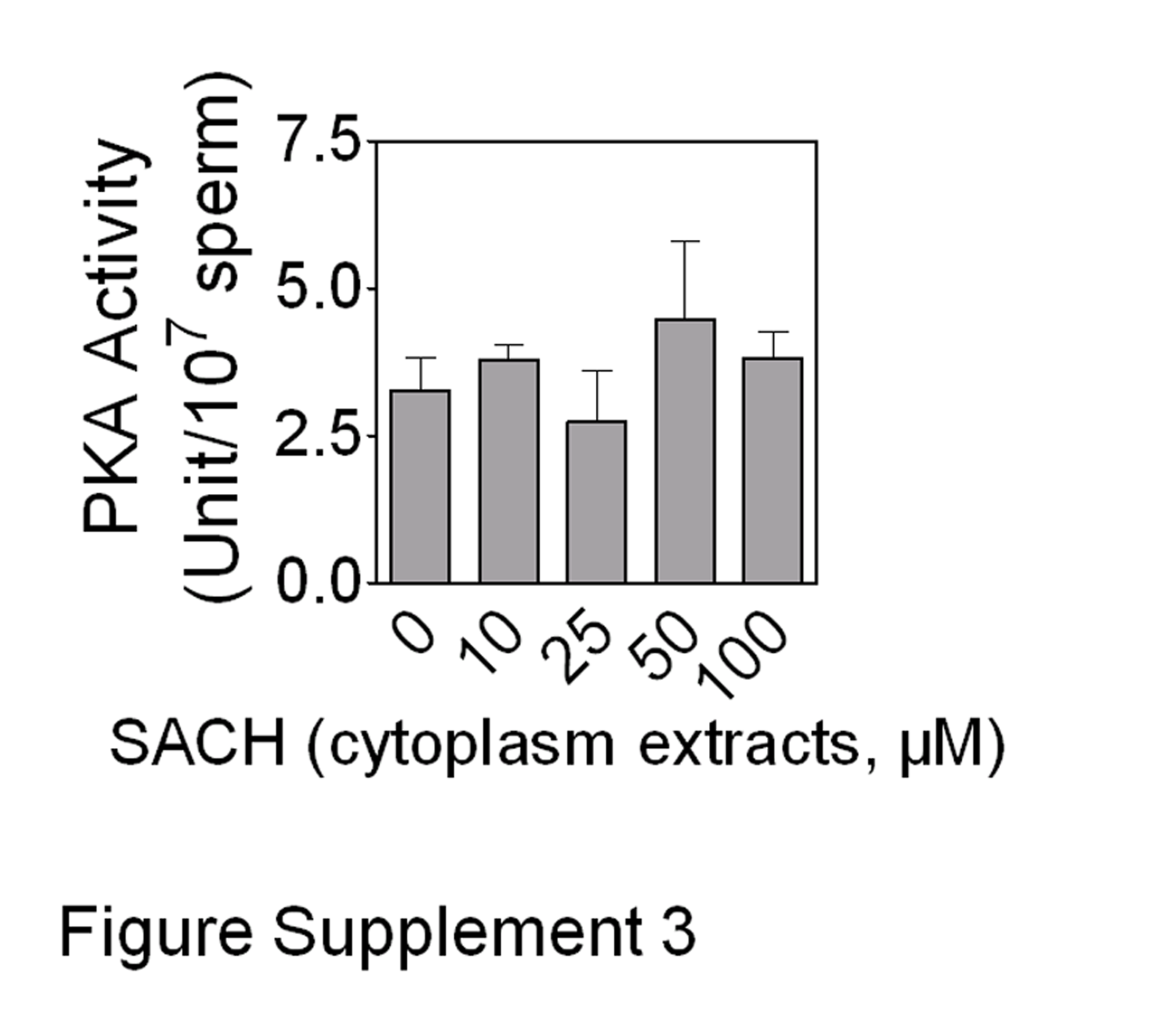

Supplement: Figure S3 — Effects of SACH on PKA activity in rat sperm extracts. Rat sperm were homogenized and the cytoplasm extracts were incubated with 10, 25, 50 and 100 µM SACH in PKA reaction mixture for 30 min, and then followed by PKA activity measurement. The reaction system without PKA was taken as negative control, and the positive control reaction system contained 0.4 µg/ml PKA catalytic subunit. Data represent as mean ± SEM, n = 3. (TIF) [file pone.0043004.s003.tif]

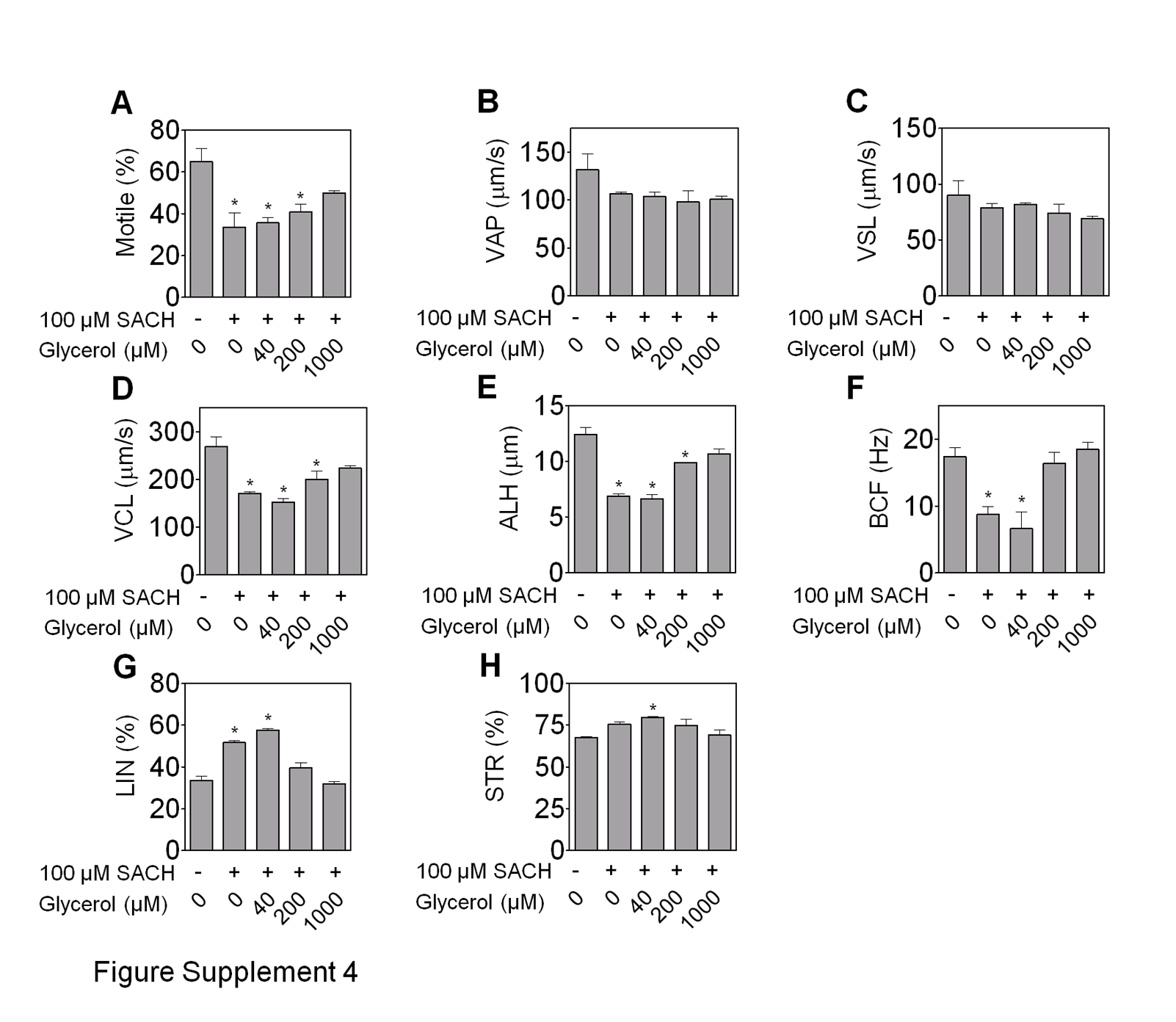

Supplement: Figure S4 — Glycerol restores rat sperm motility inhibited by SACH. Rat sperm were treated with 0, 0.04, 0.2 and 1.0 mM glycerol in the presence of 100 µM SACH for 6 hours in BWW, and CASA was performed to measure sperm kinematic parameters motile, VSL, VAP, VCL, ALH, LIN, BCF and STR. Data represent as mean ± SEM, n = 3. (TIF) [file pone.0043004.s004.tif]
